# Recalibration of the Limiting Antigen Avidity EIA to Determine Mean Duration of Recent Infection in Divergent HIV-1 Subtypes

**DOI:** 10.1371/journal.pone.0114947

**Published:** 2015-02-24

**Authors:** Yen T. Duong, Reshma Kassanjee, Alex Welte, Meade Morgan, Anindya De, Trudy Dobbs, Erin Rottinghaus, John Nkengasong, Marcel E. Curlin, Chonticha Kittinunvorakoon, Boonyos Raengsakulrach, Michael Martin, Kachit Choopanya, Suphak Vanichseni, Yan Jiang, Maofeng Qiu, Haiying Yu, Yan Hao, Neha Shah, Linh-Vi Le, Andrea A. Kim, Tuan Anh Nguyen, William Ampofo, Bharat S. Parekh

**Affiliations:** 1 International Laboratory Branch, Division of Global HIV/AIDS, Centers for Disease Control and Prevention, Atlanta, Georgia, United States of America; 2 Epidemiology and Strategic Information Branch, Division of Global HIV/AIDS, Centers for Disease Control and Prevention, Atlanta, Georgia, United States of America; 3 The South African DST/NRF Centre of Excellence in Epidemiological Modelling and Analysis (SACEMA), University of Stellenbosch, Stellenbosch, South Africa; 4 School of Computational and Applied Mathematics, University of the Witwatersrand, Johannesburg, South Africa; 5 Thailand Ministry of Public Health-US CDC Collaboration, Bangkok, Thailand; 6 National AIDS Reference Laboratory, National Center for AIDS/STD Control and Prevention, Chinese Center for Disease Control and Prevention, Beijing, China; 7 California Department of Public Health, Richmond, California, United States of America; 8 Division of Global HIV/AIDS, Centers for Disease Control and Prevention, Hanoi, Vietnam; 9 CDC-Kenya, Nairobi, Kenya; 10 National Institute of Hygiene and Epidemiology, Hanoi, Vietnam; 11 Noguchi Memorial Institute for Medical Research, Accra, Ghana; University of Rome Tor Vergata, ITALY

## Abstract

**Background:**

Mean duration of recent infection (MDRI) and misclassification of long-term HIV-1 infections, as proportion false recent (PFR), are critical parameters for laboratory-based assays for estimating HIV-1 incidence. Recent review of the data by us and others indicated that MDRI of LAg-Avidity EIA estimated previously required recalibration. We present here results of recalibration efforts using >250 seroconversion panels and multiple statistical methods to ensure accuracy and consensus.

**Methods:**

A total of 2737 longitudinal specimens collected from 259 seroconverting individuals infected with diverse HIV-1 subtypes were tested with the LAg-Avidity EIA as previously described. Data were analyzed for determination of MDRI at ODn cutoffs of 1.0 to 2.0 using 7 statistical approaches and sub-analyzed by HIV-1 subtypes. In addition, 3740 specimens from individuals with infection >1 year, including 488 from patients with AIDS, were tested for PFR at varying cutoffs.

**Results:**

Using different statistical methods, MDRI values ranged from 88–94 days at cutoff ODn = 1.0 to 177–183 days at ODn = 2.0. The MDRI values were similar by different methods suggesting coherence of different approaches. Testing for misclassification among long-term infections indicated that overall PFRs were 0.6% to 2.5% at increasing cutoffs of 1.0 to 2.0, respectively. Balancing the need for a longer MDRI and smaller PFR (<2.0%) suggests that a cutoff ODn = 1.5, corresponding to an MDRI of 130 days should be used for cross-sectional application. The MDRI varied among subtypes from 109 days (subtype A&D) to 152 days (subtype C).

**Conclusions:**

Based on the new data and revised analysis, we recommend an ODn cutoff = 1.5 to classify recent and long-term infections, corresponding to an MDRI of 130 days (118–142). Determination of revised parameters for estimation of HIV-1 incidence should facilitate application of the LAg-Avidity EIA for worldwide use.

## Introduction

Laboratory methods to detect recent HIV infection and estimate HIV incidence using cross-sectional specimens continues to be a high priority because they have the potential to help monitor the leading edge of the epidemic, target resources and evaluate successes of prevention programs in a very cost-effective and timely manner [1–18]. Measurement of HIV-1 incidence is also critical for identifying high incidence populations for prevention trials, including efficacy of candidate vaccines and other interventions.

The development of an optimal laboratory method for worldwide use has remained challenging due to the diversity of HIV-1 subtypes, biologic differences among populations or limitation of the assays [1,19–25]. Several reviews and reports have been written summarizing the status of the evolving research in this area; they have stressed the need for accurate calibration of assays or algorithms but substantive progress has been slow [4,22,26–31].

In the absence of reliable laboratory methods, UNAIDS and others have derived incidence estimates based on mathematical modeling [32–36], while others have used prevalence in younger age groups or successive rounds of prevalence to estimate incidence [37–42]. Incidence estimates based on mathematical modeling are retrospective, not timely and have their biases. Additional limitations of modeling include inability to generate subgroup and risk factor analysis which are critical for understanding current transmission dynamics and for designing prevention strategies. In addition, increasing but variable ART coverage and decreasing mortality in most countries require input of additional but uncertain parameters into models, further contributing to potential biases.

In recent years, definitive progress has been made in the identification of new biomarkers and the development of assays, including molecular methods and rapid tests to detect and distinguish recent from long-term infections [5–7,43–46]. Reliable laboratory assays, if available, are attractive because of ease of use, application to cross-sectional population, low recruitment bias, low cost and provision of real-time incidence estimates. We recently described a novel, single-well limiting-antigen (LAg) avidity assay [5]. This novel concept was further developed into an optimized assay [6] and characterized with respect to its performance in multiple subtypes. Subsequently, we have transferred the assay to two commercial entities for development of a kit and have conducted field evaluations in several populations worldwide in countries such as Vietnam, Ghana, Swaziland, and Kenya (to be published separately).

In March 2013, we organized a consultation meeting of experts to review data pertaining to characteristics, performance, and validation of the LAg-Avidity EIA. One of the recommendations included review of the mean duration of recent infection (MDRI) analysis. Although our previous report described the MDRI of 141 days at cutoff ODn of 1.0, our and others’ subsequent work indicate that the method used to determine the MDRI was not applied optimally and recalibration of the assay was needed. We describe here the revised estimates of the MDRI using data from >250 seroconverters panels at various cut-offs using multiple statistical methods to ensure that these estimates are reliable and recommend a new MDRI at a preferred cutoff for application in cross-sectional incidence estimates.

## Materials and Methods

### Specimens

Longitudinal specimens (n = 2737) from 259 individuals infected with HIV-1 were collected as part of various cohort studies in different locales by different investigators. The specimens from consenting individuals were made available to permit development and characterization of new incidence assays, including the LAg-Avidity EIA. Some of the basic information about the cohorts, including source, number of seroconverters, available specimens, and likely or confirmed HIV-1 subtypes are shown in Table 1. Of the 259 individuals, 89 of them (n = 393 specimens) were part of our previous study [6]. The following HIV-1 subtypes were included in this study: HIV-1 subtype B (Thailand BMA IDU cohort [47], Amsterdam cohort [48] and Trinidad cohort), subtype AE (Thailand BMA IDU cohort [47]), subtype C (Ethiopia and China cohorts), subtypes A & D (Kenya CSW cohort [49]). Because early antiretroviral therapy can affect the development and maturation of HIV antibodies, only specimens from persons who were not on antiretroviral therapy (ART) were used for determination of MDRI. Time between last negative and first positive specimens ranged from 4 days to 1486 days for different panels with median interval of 125 days and mean of 171 days.

**Table 1:** Country of origin, subtypes and specimens information for the seroconversion panels used in the study.

Cohort	HIV-1 Subtypes	No. of Subjects	No. of Specimens
Netherlands, Trinidad, Thailand, US	B	69	704
Thailand	AE	97	1620
Ethiopia, China	C, BC, AE	59	332
Kenya	A, D	34	81
ALL	A, AE, B, C, BC and D	259	2737

An additional 3740 specimens from treatment-naïve adult individuals with HIV-1 infection longer than 1 year were used to estimate the proportion of specimens misclassified as recent, termed here as the proportion false recent (PFR). The specimens were collected under multiple approved protocols that permitted use of left-over, unlinked specimens for research. This set included 1845 specimens from Vietnam, 952 specimens from Ghana, 455 specimens from China and 488 specimens from individuals with AIDS (CD4<200). Specimens from individuals with AIDS were derived from three sources: 261 specimens were collected in the 1990s from treatment-naïve women with AIDS enrolled in the HIV Epidemiologic Research Study (HERS) [50], while additional specimens were from Thailand (n = 128) and Cote d’Ivoire (n = 99), collected in the 1990s from treatment-naïve AIDS patients with (Cote d’Ivoire) or without (Thailand) tuberculosis (TB).

This study was conducted under a protocol approved by Centers for Disease Control and Prevention (CDC) Institutional Review Board (IRB) titled “characterization, validation and application of HIV-1 incidence assays”. Selected specimens were collected under multiple CDC approved protocols (IRB # 5533, 5758). Study was also approved by Bangkok Metropolitan Administration Ethics Committee, respective ministries of health and CDC. Individuals donating the blood specimens provided written consent for use of the specimens for biological research.

### LAg-Avidity EIA

The LAg-Avidity EIA was performed as described earlier [6]. Following successful transfer of technology to a company, commercially produced kits were used to perform the testing (Sedia BioSciences, Portland, OR). These kits were verified as having the same performance characteristics as our in-house assay, including a matching Calibrator (CAL) specimen, a key to classification of recent and long-term infection. Details of the assay steps are as follows as per manufacturer’s instructions: Assay controls [Negative control (NC), CAL, low-positive control (LPC) and high-positive control (HPC)] or HIV-positive specimens were diluted 1:101 in specimen diluent and 100 μL of controls or specimens were added to appropriate wells of antigen-coated plates and incubated for 60 min at 37°C. Controls are included in duplicate (NC) or triplicate (other controls) on each plate, while specimens were tested in singlet. Plates were washed 4 times with 1x wash buffer to remove unbound antibodies. A pH 3.0 buffer was added to each well (200 μL/well) and incubated for 15 min at 37°C to dissociate low avidity antibodies, if any. Following 4 washes, goat-anti-human IgG peroxidase (100 μL/well) was added to each well and incubated for 30 min at 37°C. Tetramethyl benzidine substrate (100 μL/well) was then added and incubated for 15 min at 25°C. Color development was stopped by addition of 100 μL/well of 1N H_2_SO_4._ The optical density (OD) was read at 450 nm with 650 nm as a reference using a spectrophotometer.

Raw OD for each specimen was normalized using CAL OD on each plate as a ratio, such that normalized OD (ODn) = (OD of specimen/median OD of CAL). For the purpose of this exercise, all specimens were tested on two independent runs in singlet and the mean ODn was used for further analysis. Plates were validated using acceptable values of OD and ODn for each control and CAL as determined for the kit. If one or more of the controls fell outside of the acceptable ranges defined in the kit insert, the run was rejected. Specimens were then re-tested and ODn values from only valid runs were used for analysis. To assist with data management and analysis, an Excel-based data management tool was developed to auto-validate each plate, calculate ODn and classify specimens as recent or long-term infections based on 1.5 cutoff.

### Statistical Methods to Determine MDRI

Formally, the Mean Duration of Recent Infection (MDRI) of an assay, a required parameter of an assay to estimate incidence, is the mean time which subjects spend classified as ‘recently infected’ during a period T post-seroconversion. The Proportion of False Recent (PFR) result is a population-level probability of obtaining a ‘recently infected’ result on a randomly chosen person infected for more than time T which was set to one year [51]. We used seven different statistical approaches to derive the MDRI for the LAg assay at various cutoffs; they are presented below. Methods 1, 3, 6 and 7 assumed that the sero-conversion occurred at the mid-point of last negative and first positive dates while for methods 2, 4, and 5 sero-conversion was assumed to have occurred at any time between the last negative and first positive dates with uniform probability.

Methods 1 and 2 (Empirical methods balancing false recent and false long-term): These two methods for estimating the mean duration of recent infection (MDRI) for the LAg Avidity assay use the “empirically balanced observation time” approach. The methods are based on some of the early work on incidence assays [3] suggesting that the rates for false-recents and false-long terms need to balance out, especially within the interval between 0 and 1 year (T = 365 days) post-serconversion. Also for method 2, ODn values were raised to the power λ = 1.53, as estimated using a repeated measures model, in order to linearize the relationship between the ODn and time values. Daily values were determined by linear interpolation between time points or extrapolation from the last two points on either the untransformed (method 1) or on the transformed (method 2) ODn^λ^ scale. The baseline ODn value at day 0 was considered to be 0.05, equivalent to the background signal on the assay. Specimens from all serconversion panels, without exclusion, were used when applying these methods. Confidence intervals were determined by 10,000 replicate MDRI estimates obtained subject-level bootstrap resampling.

Method 3 (Linear interpolation [SACEMA-1]): This approach is based on linearly interpolating the LAg ODn values between visits, per subject. Each subject is assigned a reading of 0 at infection, and there is no extrapolation beyond the last visit. Using the interpolating readings, P_R_(t), which is the probability of testing ‘recent’ at time t post-infection, is estimated by the proportion of available results below the threshold at t post-infection.

Methods 4 & 5 (Binomial regression [SACEMA-2 and -3]): A linear binomial regression is used to model P_R_ (t), as a function of time since infection, t [52]. Although the regression model does not account for the clustering of data points by subject, estimates of uncertainty through case bootstrap resampling do. The general form of the linear binomial regression model is g(p) = **β** ^T^ **x,** where p is the probability of testing ‘recent’, g(.) is the link function, and η = **β** ^T^ **x** is the linear predictor **(β** is a vector of model parameters, and **x** is the vector of predictors). Two model were fitted: (i) A two-parameter model using a loglog link, where the linear predictor is a linear function of time (SACEMA-2); and (ii) a five-parameter model using a logit link, where **x** consists of the basis functions of a natural cubic spline over [0,T], with knots occurring every two months **(**SACEMA-3). Data points more than T×110% post-infection were not used in the fitting.

For both methods, Ω_T = ᶴ___0_^TP_R_ (t) dt is then estimated using the composite trapezoidal rule for integration (20,000 subintervals). Assuming uniformly distributed infection times between last HIV-negative and first HIV-positive visits, the (expected) infection times are used (midpoints of inter-visit intervals), with no further accounting for uncertainty in infection times. Confidence interval (CI) limits are estimated by the percentiles of (1000) replicate MDRI estimates obtained by subject-level bootstrap resampling [53].

Method 6 (Nonparametric Survival Analysis): A nonparametric survival analysis method for interval-censored data was used to estimate the recency period of the assay. This approach required fewer assumptions than other approaches. It was adapted in our context where it was known that sero-conversion has occurred between two time points t_1_ and t_2_ and assay threshold was crossed between time points t_3_ and t_4_, where t_1_ ≤ t_2_ ≤ t_3_ ≤ t_4_. Therefore the recency period lies in the interval (t_3 – _t_2_) to (t_4_ – t_1_). If the threshold was not crossed by the last observation then the upper end of this interval was set as censored. These two limits were used to calculate the maximum likelihood estimate of the survival curve. The mean estimates of the recency period were directly derived from the survival curve. For the mean to be defined finitely, it was assumed that the event occurred for the longest observed subject at the latest observed time. We employed a SAS macro called EMICM to estimate the survival curve for the recency period [54]. Confidence intervals were estimated based on bootstrap techniques. Upper and lower limits of the interval were derived as 97.5^th^ and 2.5^th^ percentiles of the empirical distribution. Threshold values from 1.0 to 2.0 were used to derive MDR estimates for each of the subtypes and overall recency period.

**Fig 1:**
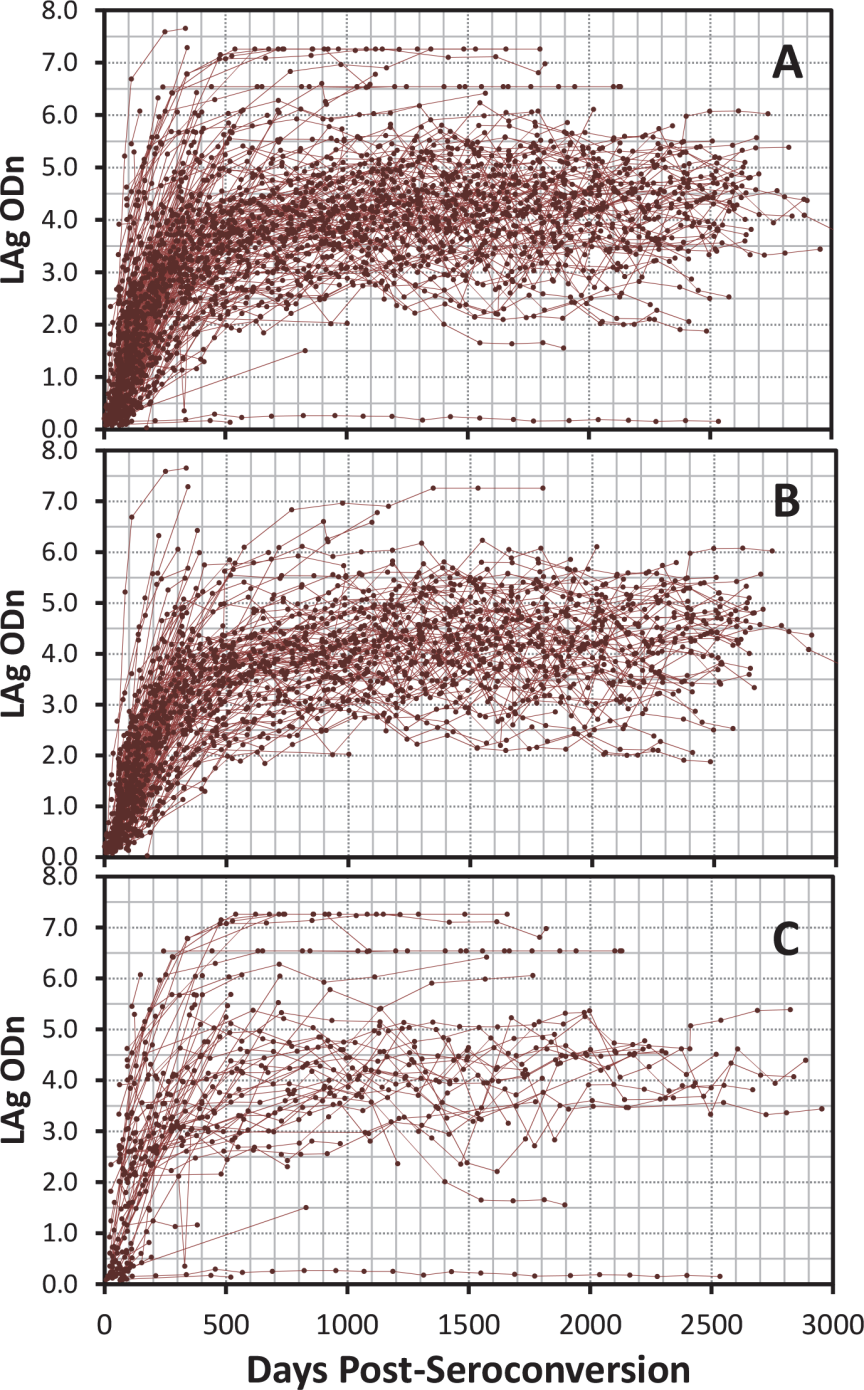
A: Changes in antibody avidity as measured by LAg-Avidity EIA post-seroconversion for all 259 seroconversion panels. For the purpose of these plots, midpoint of last negative and first positive dates for each panel was used as the seroconversion date to calculate days post-seroconversion (X-axis). B: Changes in avidity for 176 panels after exclusion of suboptimal panels as required by some methods. C: Changes in avidity as depicted for suboptimal panels that were excluded for some methods.

Method 7 (Individual Panel Regression Analysis): Seroconversion (SC) panels were included in the analysis if they exhibited a rise in ODn response over the collection period and a regression equation could be fitted describing the antibody avidity kinetics. A total of 176 optimal SC panels comprising 2076 specimens were included in this analysis (Fig. 1B). Midpoint of last negative and first positive dates was used as the seroconversion date which was designated as day 0 and used to calculate days since seroconversion for subsequent longitudinal specimens. Each individual SC panel was plotted and a regression equation was generated using Excel. The regression equation was then solved for the desired cutoff value to determine estimated the number of days it required for that individual to reach the LAg assay cutoff. The mean duration of recent infection was then calculated by averaging the all SC panel results, along with the 95% CI for the mean. The mean duration of recent infection was calculated by subtype and overall, considering all subtypes. While the data requirements for this method are minimal, it does not use all the available data, such as the plateau data points which were not fully utilized.

### Determination of Proportion False Recent (PFR)

The PFR was determined as % of specimens collected more than one year post-seroconversion, which were misclassified as recent HIV-1 infection by the LAg-Avidity EIA. The PFR was calculated at each ODn cutoff of 1.0, 1.25, 1.5, 1.75 and 2.0 to evaluate the extent of misclassification at the various cutoffs. The 95% confidence intervals were calculated for each PFR.

## Results

### Antibody Avidity Kinetics

Antibody avidity maturation, as measured by LAg-Avidity EIA, for all 259 seroconverters is shown in Fig. 1A. Overall, there is an increase in avidity of gp41-specific antibodies following seroconversion, reaching a plateau level at about 500 days post-seroconversion. Most individuals exhibited normal increase in avidity kinetics; however, a few individuals showed some decline in antibody avidity over time while in rare cases antibody avidity remained low. Since statistical methods to calculate the MDRI can be affected by the duration between last negative and first positive results, collection interval, avidity kinetics and/or frequency of specimen collections, we separated optimal and sub-optimal seroconverter specimen sets. Fig. 1B shows 176 seroconverters (2076 specimens) with optimal time interval between last negative and first positive specimens (<100 days), with 3 or more specimens per donor with regular collection schedules, and also exhibiting a typical rise in antibody avidity levels. Fig. 1C shows 83 individuals (641 specimens) with one or more of the following when examined individually: sub-optimal collection schedules, longer time interval between last negative and first positive specimens (>100 days), or atypical antibody kinetics not crossing the potential cut-off threshold of ODn 1.0 to 2.0. It is interesting to note that when examined collectively, the antibody kinetics in Fig. 1C are not very different from those in Fig. 1B, except in rare cases when antibody avidity remained low, a likely contribution from elite controllers.

### MDRI and PFR Results

Analysis of the data by 7 different statistical methods for calculation of MDRI at varying cutoffs between ODn of 1.0 and 2.0 are summarized in Table 2. Overall, the different methods provided similar results at each given cutoff. For example, at cutoff of 1.0 ODn, seven methods yield an MDRI of 87 to 94 days, while at cutoff of 1.5 ODn, the MDRI varied from 130 to 137 days. Corresponding misclassification (proportion false recent, PFR) among 3740 individuals with true long-term infections (>1 year) are shown in the right column (Table 2). This PFR represents overall misclassification frequency for all specimens, irrespective of subtypes or geographic locations. The PFR increased from 0.6% to 2.5% when threshold cutoff increased from 1.0 to 2.0 ODn, respectively. Subtype or country specific PFR data will be further analyzed in separate reports.

**Table 2:** Summary of 7 different methods used for determination of mean duration of recent infection (MDRI, in days with 95% CI) at varying cutoffs on LAg-Avidity EIA with corresponding level of proportion false recent (PFR) classification.

MDRI Results of Different methods (1 to 7) Used								
Cutoff ODn	1	2	3	4	5	6	7	PFR (%)
1	91 (83–99)	87 (76–98)	92(79–104)	89 (81–98)	88 (78–98)	88 (79–98)	94(85–103)	0.6
1.25	114 (106–125)	110 (98–124)	113 (102–123)	110 (100–120)	110 (99–122)	109 (98–121)	112 (102–123)	1.0
1.5	137 (127–150)	133(121–149)	136 (122–150)	130 (119–141)	132 (120–144)	130 (118–142)	132 (121–143)	1.6
1.75	160 (150–174)	156.0 (143–173)	149 (135–164.0)	151(139–162)	147 (120–144)	145 (132–158)	155 (142–168)	1.9
2	183 (169–197)	177 (163–195)	171 (154–189)	171 (158–184)	166 (152–180)	161 (148–174)	181 (166–196)	2.5

See Methods section for further details of different methods used*.

*Methods 1 = Balanced time & 0,w and w,365, midpoint SC; 2 = Balanced time & 0,w and w,365, uniform SC; 3 = Non-Parametric MLE; 4 = Linear Interpolation; 5 = Binomial Regression (A); 6 = Binomial Regression (B); 7 = Individual SC Panel Regression. See Methods section for further details.

Differences of MDRI among different methods were minimal. Demonstratively, method 6, a binomial regression method that utilized all data points, indicated that at cutoff of 1.0 ODn, the MDRI was 88 days (95% CI 79–98) with a corresponding PFR of 0.6%, while at cutoff of 1.5 ODn, the MDRI was 130 days (95% CI 118–142) with a corresponding PFR of 1.6%. The MDRI increased to 161 days (95% CI 148–174) at cutoff of 2.0 ODn but there was a corresponding increase in PFR to 2.5%.

**Fig 2:**
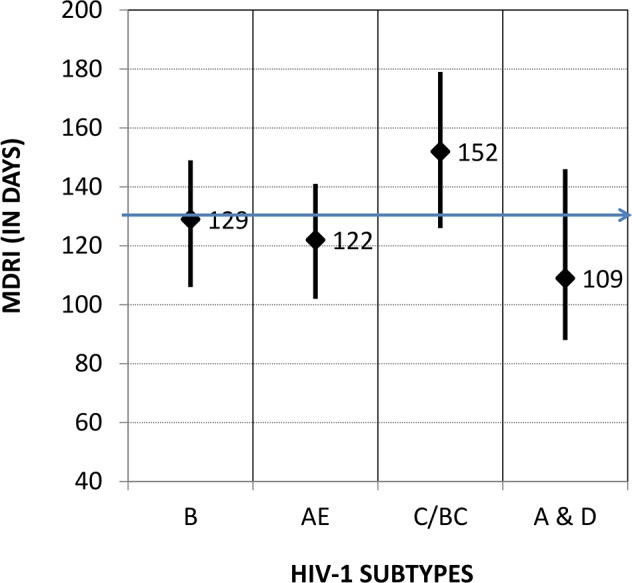
Mean duration of recency at cutoff ODn of 1.5 by subtype or geographic region as determined by bionomial regression method that uses all specimens without any exclusion criteria. Closed diamonds represent MDRI for different subtypes and vertical lines represent upper and lower 95% CI. The horizontal line represents overall mean MDRI of 130 days.

Fig. 2 shows MDRIs by different subtypes or by geographic location (e.g., A&D from Kenya) using Method 6. MDRIs by subtypes were: 129 days (subtype B), 122 days (subtype AE), 152 days (subtype C) and 109 days (subtypes A&D). Although there are some differences, use of an overall MDRI of 130 days (horizontal arrow) is appropriate for application to determine HIV-1 incidence in cross-sectional populations.

**Fig 3:**
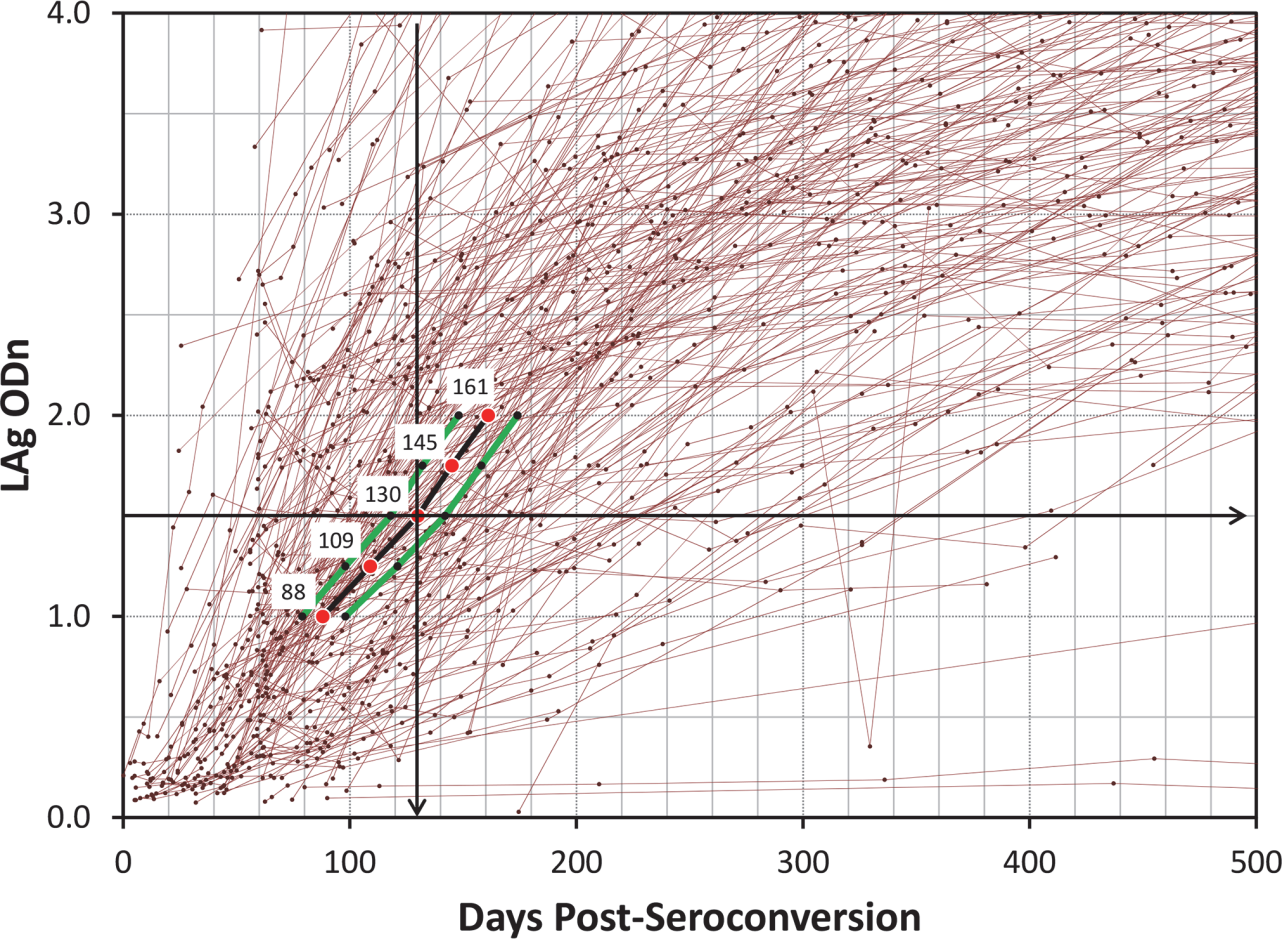
Close-up view of changes in avidity with overlap of MDRI at cutoffs of 1.0 to 2.0 as determined by binomial regression method. The red lines represent 95% bounds around MDRI.

We examined the calculated MDRIs (Method 6) at different cutoffs overlapping with the avidity kinetics during the early period of seroconversion (<500 days) as shown in Fig. 3. The line joining the MDRIs at varying cutoff goes through the middle portion of the increasing avidity in this close up view of antibody maturation. The 95% CI around the MDRIs are indicated with the red lines.

## Discussion

The MDRI is an essential characteristic of an incidence assay for appropriate application of the assay in cross-sectional estimation of the HIV-1 incidence. We previously reported optimization and characterization of LAg-Avidity EIA [6], including the determination of the MDRI using longitudinal specimens from 89 seroconverters. However, further review of the data indicated that the method used to calculate the MDRI was not properly applied to the dataset, resulting in an overestimate of the MDRI for the LAg-Avidity assay at the cutoff 1.0 ODn. Application of an elevated MDRI would result in an underestimation of HIV incidence if applied to a cross-sectional cohort. Therefore, this recalibration exercise was necessary and in order to achieve a more representative and robust estimate, we increased the number of seroconversion panels from 89 to >250 panels that represent more diverse subtypes and included multiple statistical methods to ensure accuracy and consensus of the final results.

In all, we used seven different statistical methods to determine the MDRI values and found that these methods gave very similar results providing further confidence to the robust nature of the analysis and the methods employed (Table 2). Our new results show that the MDRI of the LAg-Avidity EIA using the highlighted binomial regression method was 88 days and 161 days at the cutoffs of 1.0 to 2.0 ODn, respectively (Fig. 3). Determination of the optimal cutoff for cross-sectional application is a balance between MDRI (which should not be too small) and PFR (which should not be too large)[27,31,55,56]. At the cutoff of 1.5 ODn, our overall PFR was 1.6%, lower than 2% recommended by WHO Incidence Working Group [57] for new incidence assays. A cutoff of 1.5 ODn provides this balance between duration of MDRI (130 days) and PFR (<2.0%) using specimens in our collection. Therefore, we propose a default cutoff of 1.5 ODn to classify recent and long-term infections; this represents the mean duration of 130 days (95% CI 118–142) since seroconversion. It is recommended that the studies conducted previously with the LAg-Avidity EIA should reanalyze their data using revised cutoff (ODn<1.5) for recent HIV infection classification and MDRI of 130 days as per our new analysis. This revision does not impact the raw data generated using the LAg-Avidity EIA, just the interpretation and use of the data.

Statistical methods used to determine MDRIs have varied since the first description of the detuned assay in 1998 [9]. Since then multiple approaches have been used, partly due to lack of consensus among statisticians about the best methods [3,11,15,18,21,46,58]. Under the auspices of WHO Incidence Working Group, a statistical workshop was organized in 2011 to develop a consensus and promote a preferred method(s). Although there was some broad agreement and a better understanding of various approaches used, differences in the approaches remain, and no detailed benchmarking has been carried out. Recently, a benchmarking project has been launched under the auspices of the HIV Modeling Consortium (funded by the Bill and Melinda Gates Foundation) with a focus on identifying strengths and limitations of a diverse range of methods. Our analyses show that when used appropriately with a robust dataset, several of these methods yield comparable estimates of MDRIs.

Comparison of the MDRIs by HIV-1 subtypes/population show that the MDRIs varied from 109 days (subtype A&D) to 152 days (subtype C). Further evaluation of the MDRI in more seroconversion panels collected from individuals infected with divergent HIV-1 subtypes and geographic locations should provide further data on subtype differences, if any. Such assessment by independent groups, such as CEPHIA, will be critical for this and other incidence assays. If subtype-specific differences are confirmed, use of MDRI for prevalent subtype is appropriate and may be considered when applying the LAg-Avidity EIA keeping in mind that trend of HIV incidence measured over time is more important than a single point estimate to assess the impact of HIV prevention efforts.

Although the PFR of 1.6% at the 1.5 ODn cutoff was below the recommended level of <2% in our study, this PFR was determined in ART-naïve populations. We realize that the actual PFR will vary in different populations depending on the state of the HIV epidemic, overall ART coverage, timing of ART initiation and duration of ART. Early initiation of treatment before maturation of antibodies will prevent the development of high-avidity antibodies and will result in misclassification of long-term infections on most antibody-based assays, including LAg-Avidity EIA (unpublished data). Collection of additional clinical information about ART use during the surveys can help address this issue.

The continuing need to determine the local PFR during each round of survey is a burden for surveillance systems and can be impractical. False-recent classifications are caused primarily by elite controllers or individuals on treatment. Both of these cases can be effectively identified by testing for viral load, such that the LAg recent samples with VL <1000 copies/mL, for example, would be classified as long-term. This approach is attractive for multiple reasons 1) it reduces the need to conduct exhaustive PFR studies, 2) it identifies misclassified elite controllers and those on ART in the study pool, and 3) it improves accuracy of incidence estimates. Given that this testing will be done only on LAg-recents (usually <10% of total positives) and many national reference laboratories in developing countries can now perform viral load testing, this is logistically feasible.

Recently, a multi-assay algorithm (MAA) has been suggested that includes 1) BED-capture EIA at a higher cutoff as the first step, followed by 2) Bio-Rad Avidity EIA, again at a higher cutoff 3) then CD4 measurement, and 4) finally VL measurement to classify recent HIV infection[52,59,60]. It is unclear how each of the multiple components of this somewhat complex algorithm contributes, whereas the relative simplicity of a single immunoassay and VL may suffice to provide sufficient precision. Additionally, any algorithm using CD4 will have limited application in surveys that collect dried blood spot (DBS) specimens for incidence testing. It should be pointed out that there are on-going developments in the area of HIV incidence algorithm development.

For wider application of the LAg-Avidity EIA to estimate HIV incidence, the next steps will include field validation of the revised parameters in cross-sectional populations and comparison of the LAg-derived estimates with other reference estimates of incidence in the same population. Further association of demographic and other risk factors in the context of HIV-1 incidence and prevalence should further assist in validation of the assay.

In summary, we have recalibrated the LAg-Avidity EIA using >250 longitudinal seroconversion panels from multiple subtypes derived from diverse geographical locations using several methods. Based on these data, we recommend a cutoff ODn of 1.5, which corresponds to an MDRI of 130 days (95% CI 118–142) for application in a cross-sectional population for estimation of HIV-1 incidence and risk-factor analysis. Determination of these parameters and recent availability of the assay kits from two manufacturers (Sedia BioSciences and Maxim BioMedical), including a dried blood spot (DBS) kit from the latter manufacturer, should further facilitate measurement of HIV-1 incidence in cross-sectional populations for program planning and impact evaluation of prevention and intervention efforts worldwide.

## Supporting Information

S1 Table Spreadsheet with LAg-Avidity EIA data on seroconversion panels (donors = 259; specimens = 2737) from multiple countries as described in Methods section. Mean ODn of two independent runs was used for this analysis.
(XLSX)

S2 Table Spreadsheet with LAg-Avidity EIA data on specimens from individuals (n = 3740) with known long-term infections (>1 year) for the purpose of determining proportion of false recent (PFR) classification. Mean ODn of two independent runs was used for this analysis.
(XLSX)
